# Sleeve gastrectomy without bougie is safe and effective operation: case report

**DOI:** 10.1093/jscr/rjaa183

**Published:** 2020-06-19

**Authors:** Zkria Atia Shekh, Abdulkafi Hasan Roqaia

**Affiliations:** 1 Department of General Surgery, Syrian Private University, Damascus, Syria; 2 Department of General Surgery, Damascus University, Damascus, Syria

## Abstract

Sleeve gastrectomy (SG) is an effective method for weight loss; it is done by laparoscopy or open approach; it is a restrictive procedure and involves removing part of the stomach by cutting over bougie, which ensures that stenosis doesn’t occur. Bougie may cause esophageal perforation. There are studies that show the size of bougie used and weight loss are related. We show a case of open SG without using bougie at all. It is an effective and safe method and results in ~30% of weight loss 3 months after the operation without complications; thus, we conclude that SG can be done without bougie.

## INTRODUCTION

Sleeve gastrectomy (SG) is one of bariatric surgeries, which is a very popular procedure; it is a very effective operation for weight loss; it is usually performed laproscopically and can be carried out by open approach [1]. SG involves removing most of the stomach body; this is restrictive procedure and removes ghrelin-producing site, which is responsible for appetite [1, 2]. Ghrelin is a 28-amino acid peptide predominantly secreted by the oxyntic glands of the proximal part of the stomach with lesser amounts produced by the bowel, pancreas and hypothalamus [1].

Bougie is usually used to remove stomach tissue without causing stenosis. SG usually leads to loss of ~50%of body weight within 6 months [3]. It leads to weight loss in two ways: it restricts the size of the stomach and decreases circulating levels of ghrelin [1].

We show a case of SG carried out by open approach and cutting stomach by estimation without bougie, and this is a safe and effective method.

## CASE PRESENTATION

This is a case of 25-year-old woman of body mass index (BMI) of 36 with comorbidity (sleep apnea) and was indicated for SG after medical therapy had failed [2]. The patient was poor and can’t pay for staplers used in laparoscopic SG, so we proceeded with open SG by midline incision ~10 cm above the umbilicus, and after cutting omentum and mobilization of the stomach, we applied non-crushing forceps and cut the stomach among the lesser curvature without applying bougie by estimation and then sutured the stomach remnant by double layers of vicryl leaving nasogastric tube (NGT) and drain and closed the abdomen and skin intradermally. At night, NGT was removed and the following morning she started to drink clear fluids. Drain was removed on the second day.

She was followed up for 6 months to monitor weight loss during the first month, she did well and complained slightly of vomiting after heavy meals, and after 3 months, she lost ~30% of total body weight.

## DISCUSSION

SG is a popular procedure for weight loss, it is an effective method to reduce weight after non-surgical methods failed. Patients with a BMI > 40 or >35 with comorbidities of obesity are eligible for bariatric surgery, according to the National Institutes of Health consensus guidelines [1, 2]. SG is performed by laparoscopy or laparotomy. The procedure depends on removing about two-thirds of the stomach over bougie, which ensures that stenosis doesn’t occur. The bougie is used to ensure correct size of the gastric tube is a part of the standard operation and usually placed by the anesthesiologist and with a very low rate of complications [4], but it can cause esophageal perforation [5]. In our case, we didn’t use bougie at all, and estimation has been carried out by removing the stomach as much as possible without inducing stenosis, which can lead to vomiting later.

This method is discovered to be good and not affect the result of weight loss; the patient had lost ~30% of total body weight within 3 months and this was ideal.

This operation is considered clean-contaminated, so we used antibiotics prophylactic before incision and then we sutured the skin intradermally, and cosmetic result achieved without complications.

The surgeon must remove the stomach as much as possible including the fundus, the most powerful site of ghrelin production. Ghrelin stimulates appetite [6].

In our patient, we cut the stomach by estimation without bougie, and she lost appetite as typically desired, and stenosis didn’t occur, and ideal body weight loss was achieved, and those are the gold standard goals, which means that this method is safe and effective.

## CONCLUSION

SG by estimation without using bougie is a safe and effective method and can be done among open or laparoscopic operations.
